# Quantitative transcriptomic and epigenomic data analysis: a primer

**DOI:** 10.1093/bioadv/vbae019

**Published:** 2024-02-10

**Authors:** Louis Coussement, Wim Van Criekinge, Tim De Meyer

**Affiliations:** Department of Data Analysis and Mathematical Modelling, Ghent University, Ghent, 9000, Belgium; Department of Data Analysis and Mathematical Modelling, Ghent University, Ghent, 9000, Belgium; Department of Data Analysis and Mathematical Modelling, Ghent University, Ghent, 9000, Belgium

## Abstract

Summary: The advent of microarray and second generation sequencing technology has revolutionized the field of molecular biology, allowing researchers to quantitatively assess transcriptomic and epigenomic features in a comprehensive and cost-efficient manner. Moreover, technical advancements have pushed the resolution of these sequencing techniques to the single cell level. As a result, the bottleneck of molecular biology research has shifted from the bench to the subsequent omics data analysis. Even though most methodologies share the same general strategy, state-of-the-art literature typically focuses on data type specific approaches and already assumes expert knowledge. Here, however, we aim at providing conceptual insight in the principles of genome-wide quantitative transcriptomic and epigenomic (including open chromatin assay) data analysis by describing a generic workflow. By starting from a general framework and its assumptions, the need for alternative or additional data-analytical solutions when working with specific data types becomes clear, and are hence introduced. Thus, we aim to enable readers with basic omics expertise to deepen their conceptual and statistical understanding of general strategies and pitfalls in omics data analysis and to facilitate subsequent progression to more specialized literature.

## 1 Introduction

For long, the main bottleneck in molecular biology research was data generation. Individual assays for nucleic acid studies, typically based on hybridization or PCR, allowed for the characterization of single loci, leading to a linear relationship between work load and number of loci under scrutiny. Upscaling was successfully achieved by massive parallelization of the individual assays, first by means of microarrays, and later through “second generation” sequencing (Brenner *et al.* 2000, Shendure *et al.* 2005). Both technologies have matured, and though microarrays remain very valuable for specific applications, e.g. human DNA methylation studies (Bibikova *et al.* 2009), the rapid decrease in cost of high-throughput sequencing is currently making the latter technology the standard workhorse for most quantitative applications, such as transcriptomics (Van Verk *et al.* 2013) and epigenomics (Mensaert *et al.* 2014). More recently, further technical advancements have allowed for transcriptomics (Stegle *et al.* 2015) and epigenomics (Schwartzman and Tanay 2015) at the single cell level.

Whereas massive parallelization revolutionized molecular biology, it also has major implications for data analysis even beyond the large amounts of data/variables to be analyzed: (i) parallelization entails that all reactions take place under the same—for some loci unfavorable—conditions; (ii) the complexity of the process easily introduces batch differences between samples and/or subsets of samples, leading to the need for normalization; (iii) though the cost per locus is low, overall costs are rather high leading to often minimalistic study designs or low-resolution data. Evolution to single cell analyses further aggravates these issues. With this manuscript, we aim to provide a generic overview of omics strategies to address these problems and discuss associated pitfalls and related issues inherent to omics analyses, as such a more general overview is currently lacking from literature. For conceptual purposes, we explain the different steps through a generic data-analytical pipeline. However, as the latter should not *per se* be considered as the “gold standard”, we also elaborate on alternative strategies within the relevant sections.

Given the generic aim of this manuscript, we do not elaborate on each individual experimental omics technology, but refer to existing review or proof of concept articles that provide the necessary background information, e.g. for sequencing both performed on bulk data (Wang *et al.* 2009, Van den Berge *et al.* 2019) and single cell data (Chen *et al.* 2019, Luecken and Theis 2019), array based (Schulze and Downward 2001) transcriptomic data, array based DNA methylation data (Infinium HumanMethylation beadarrays) (Sandoval *et al.* 2011), bisulfite-sequencing for DNA methylation studies (Meissner *et al.* 2005, Adusumalli *et al.* 2014) and sequencing-based enrichment data [e.g. from ChIP-seq, affinity based enrichment, Cut&Tag and open chromatin assays such as (single cell) ATAC-seq] (Furey 2012, Kaya-okur *et al.* 2019, Grandi *et al.* 2022). Similarly, in line with our focus to provide conceptual insight into problems and solutions, we do not elaborate on the quality measures per technology, and refer to the major international efforts by particularly the MAQC/SEQC consortium (see https://www.fda.gov/science-research/microarraysequencing-quality-control-maqcseqc/maqc-publications, for all publications). Finally, it should be noted that some of the aspects discussed in this manuscript are also valid for DNA based molecular research (“genomics”), yet given that the data analytical workflow to screen for the presence or impact of mutations or polymorphisms is largely different from that for quantitative applications (transcriptomics and epigenomics, including open chromatin assays), we do not elaborate on this field. Likewise, a similar evolution occurred beyond nucleic acids, leading to e.g. proteomics, metabolomics and glycomics. Despite comparable problems and solutions (Putluri *et al.* 2011, Langley and Mayr 2015) and possible integration (Cavill *et al.* 2016), we considered inclusion of these omics fields beyond the scope of this manuscript.

In summary, this manuscript focuses at providing a generic strategy, associated assumptions and common pitfalls specifically relevant for quantitative (pseudo)bulk transcriptomic and epigenomic data analysis. Rather than listing and discussing all available methods, we aim at providing insight by fitting a major part of the state of the art methods in the same conceptual workflow. We start with describing relevant data resources to continue with different data analytical steps. Typically, bioinformatic tools and related preprocessing steps will be introduced here as well, but predominantly to consider the impact on subsequent statistical analysis. With this manuscript we aim to provide a deeper insight for readers with basic familiarity with transcriptomic and epigenomic data analysis and to facilitate them to progress to an expert level for making robust data analytical choices.

## 2 Generic quantitative omics data analytical workflow (Fig. 1)

**Figure 1.**
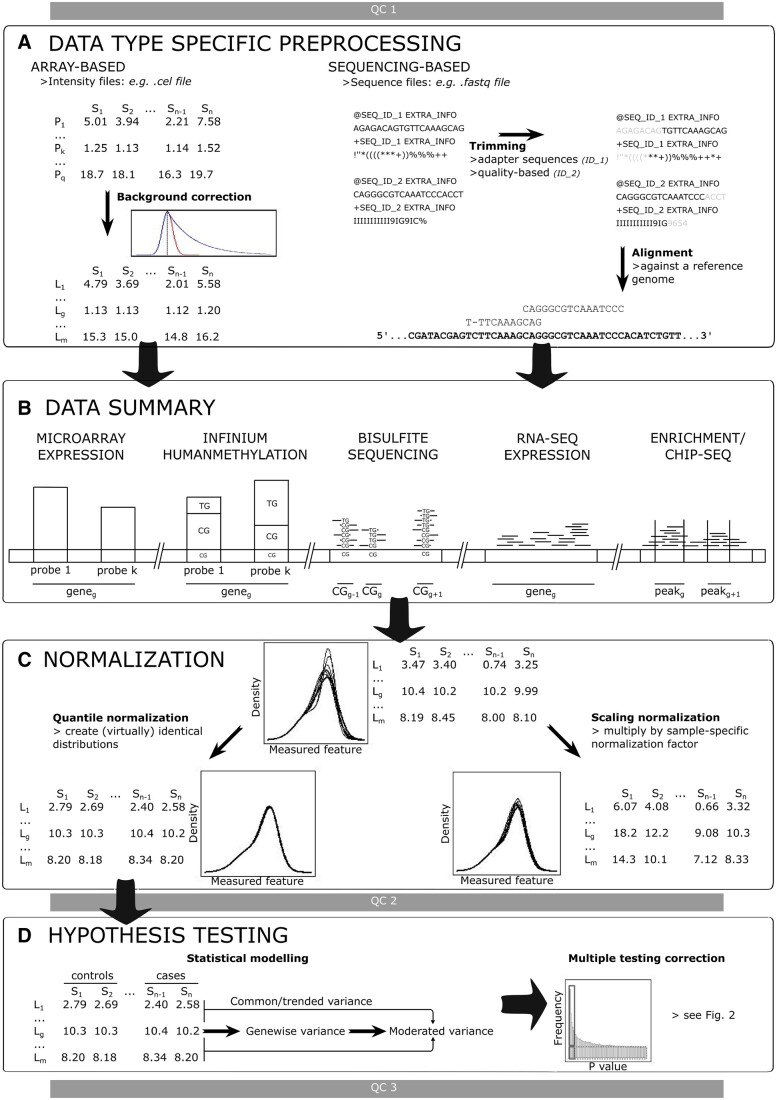
Generic omics data analytical workflow for quantitative transcriptomic and epigenomic experiments. The workflow consists of (A) data specific preprocessing, (B) data summary, (C) normalization, and (D) hypothesis testing. (A) Data specific preprocessing is performed for array based technologies, where intensity values obtained from image analysis are background corrected, and for sequencing based methods, where reads in FastQ files are aligned to a reference genome upon trimming (of low quality fragments, adapter sequences, …). (B) Data summary mostly depends on the underlying biology. In general, intensity values or mapped reads are summarized into features based on existing annotation (most often a gene, transcript, exon, CpG, …) or peak/enrichment detection procedures (e.g. for ChIP-seq data). Data summarization is here depicted on a bar which represents a genome, note that the used scale between different features might not correspond to actual sizes (e.g. CpG dinucleotide versus gene). (C) After data summary continuous (arrays, sequencing) or discrete (sequencing) data is obtained for each sample (continuous data depicted). Technical differences between samples are removed (“normalization”) to obtain more similar (scaling normalization) or even virtually identical (quantile normalization) distributions. (D) For hypothesis testing in omics analysis, typically methodologies allowing for moderation are used, i.e. “locus-wise” variance estimates are combined with the “common” variance estimate (over all loci) to obtain more robust “moderated” variance estimates. Hypothesis testing may further benefit from sample weighting and modeling of the mean-variance trend. Typically, after hypothesis testing, a correction for multiple testing is performed (see Fig. 2 and respective section in main text). Throughout the workflow multiple steps of quality control are performed using appropriate software and/or plots (e.g. FastQC for expression data). Abbreviations used in figure: QC, Quality Control; P, Probe; L, Locus; S, Sample; CG, CpG dinucleotide. Subscripts used in figure: k, kth probe; q, total number of probes; g, gth feature; m, total number of features; n, total amount of samples.

Despite major differences in experimental characteristics, both transcriptomic and epigenomic data can be analyzed with the same conceptual workflow (depicted in Fig. 1 and described in accompanying Table 1). To maintain the overall flow of the text, several options specifically aiming at improving robustness and power are discussed separately in the last subsection of this workflow. In Supplementary Information S1, we list important public data resources, given their major significance as starting point for many omics studies.

**Table 1.** Overview table accompanying Fig. 1, including all data analytical steps of the generic workflow and a short description of each step.

Data analytical step	Short description
QC1	Quality control step specific to the used methodology. This step typically has the goal of identifying samples that are featuring artefacts or problematic batch effects.
Data type specific (pre)processing	Processing of “raw data” from the platform specific to the used methodology, e.g. background correction for array-based data or trimming and subsequent alignment for sequencing based data.
Data summarization	Summarization of raw data to the feature of interest, e.g. gene, transcript, exon, peak, methylation region and individual CpG for downstream analysis. Data summarization results in a data table that has the feature of interest on its rows and the different samples that are analysed in its columns. This data table will be central and the main subject of downstream analysis.
Normalization	Normalization is performed with the goal of removing technical variance between samples (while retaining biological variance), i.e. to make samples comparable to each other. Normalization is thus performed on the columns of the data table. Normalization can be performed using different strategies with varying assumptions (see Supplementary Table S1).
QC2	Besides having a similar goal as QC1, this QC steps aims at identifying issues that arise or are created during the complete preprocessing procedure. Note that although specific plots and tools exist for separate parts of preprocessing, QC is typically performed all at once at the end of preprocessing.
Hypothesis testing	In general one hypothesis exists per feature (cf. rows of the data table). Hypothesis testing is accomplished by the use of a statistical model, typically relying on moderation to prevent type I error inflation in omics analysis.
QC3	QC step with main goal of validating results obtained in statistical analysis, i.e. sanity checks and validation of assumptions, e.g. *P*-value distribution.
Multiple testing correction	Correction for statistical testing of a large number of genes at the same time. Typically, the false discovery rate is used, since the family wise error rate is overly conservative for the typical goals of omics research.

### 2.1 Data type specific (pre)processing (Fig. 1A)

The first step of any omics workflow is data preprocessing, i.e. to process the data in such a way that it can be used for statistical analyses. Several features are common between data types, yet the first preprocessing steps tend to be different between data types. Readout from measuring equipment and transformation to a comprehensible format is typically performed using manufacturer specific software [e.g. image analysis translates array scans into probe specific values; or processing flow cell images during Illumina’s “sequencing by synthesis process” results in sequence reads with associated phred-scaled quality scores in so-called FastQ files (Cock *et al.* 2009)]. Public repositories typically store this type of preprocessed data, rather than the vast raw (image) data files. Note that although image processing has already been performed, array intensities and FastQ files are often referred to as “raw data”. For further data type specific processing we make a distinction between (i) sequence based methods (starting from FastQ files) and (ii) array based methods (starting from probe intensities).

As depicted in Fig. 1, a first round of quality control is typically performed on this “raw data”. This first quality control step is typically used to exclude sample(s) of major inferior quality or to identify unexpected batch effects (with a possible impact on conclusions) early on in the analysis. Removing suboptimal samples from further analysis of course always involves a tradeoff between obtaining higher quality data and retaining sufficient samples/power. For sequencing based methods, given its broad applicability and ease-of-use, fastQC (Andrews 2010) has become the standard for quality control of raw and preprocessed data. For microarray data, there are more commonly used approaches, however some interesting all-in-one packages exist that combine most relevant QC plots, e.g. arrayQualiltyMetrics (Kauffmann *et al.* 2009) (also including QC plots from QC2, see below). Examples of data type specific QC plots can be found in Supplementary Fig. S1A–C. These figures (and other QC figures) were created using different versions of ArrayQualityMetrics and FastQC and were based on data of published work by De Vos *et al*. (2021) and Ross *et al*. (2003), as well as unpublished data). Single cell data requires several additional QC steps (Hong *et al.* 2022), particularly to exclude droplets/wells featuring no or more than one cell from further analysis and to remove cells characterized by unsuccessful nucleic acid amplification, lack of barcode integration, ambient RNA contamination or abnormally high fractions of mitochondrial DNA, yet are not further discussed here given their less generic character.

FastQ files are further processed by alignment to a reference genome or transcriptome. Most aligners were originally designed for DNA data, e.g. BWA (Li and Durbin 2010) and BOWTIE2 (Langmead and Salzberg 2012), and further modified/extended to fully take into account the impact of splicing (RNA-seq data), e.g. TopHat (Trapnell *et al.* 2009), or of the bisulfite treatment in DNA methylation studies, e.g. Bismark (Krueger and Andrews 2011). More recently, several methods have been specifically developed for RNA-seq data, such as STAR (Dobin *et al.* 2013) and KALLISTO (Bray *et al.* 2016), with the latter providing transcript quantification without explicit alignment (“pseudoalignment”). Most aligners designed for bulk sequencing data can also be applied on single cell data (Chen *et al.* 2019), possibly enveloped by platform specific software suites, e.g. CellRanger for 10X Genomics (Zheng *et al.* 2017). The generic output format for aligned data is called the sequence alignment/map format (SAM) (or BAM for the non-human-readable binary variant allowing for more efficient computer processing) (Li *et al.* 2009), with several variants such as pseudoBAM for the output of pseudoaligners.

Note that more efficient alignment retains more read data, leading to more power for subsequent statistical analyses. On the other hand, inaccurate alignment may lead to erroneous conclusions, e.g. by associating a phenotype with a homologue rather than the genuinely involved locus. Duplicate fragments (i.e. aligning to exactly the same position in the genome) are often removed, given that they are typically the result of library preparation (which involves amplification of the starting material) and hence do not provide independent evidence for relevance of the loci involved. The main exception is bulk RNA-seq, where duplicate reads are to a certain extent expected (e.g. same transcription start site or 3’ end) and therefore most often retained. For single-cell RNA seq, the low starting amounts of RNA per cell easily lead to large amounts of duplicates upon amplification. Hence unique molecular identifiers (UMIs) are added during the first reverse transcription step and used afterwards to remove duplicate reads per cell (Zheng *et al.* 2017, Hagemann-Jensen *et al.* 2020).

Array probe intensities on the other hand do not require alignment, given that probes were designed to target known parts of the genome, yet are typically subjected to a background correction step. For expression microarrays, e.g. Affymetrix developed algorithms such as MAS5 (MicroArray Suite v5.0; https://www.shorturl.at/uPQZ4, 15 December 2022) which use the Affymetrix specific “mismatch probes” to correct the intensities of corresponding “perfect match” probes. On the other hand, a more commonly used generic method, implemented in the RMA (robust multi-array average) preprocessing pipeline (Irizarry *et al.* 2012), only models the distribution of the “match” probe intensities to separate the (exponential) signal distribution of interest from the strict-positive Gaussian noise distribution. Similar strategies have been implemented for Infinium HumanMethylation beadarrays (Triche *et al.* 2013).

### 2.2 Data summary (Fig. 1B)

Following background correction or alignment, data summary—also known as data aggregation—is the next step for omics data analysis. Data summary refers to the integration of information into variables, e.g. of reads or probes per gene for gene expression data, peak calling for enrichment sequencing data. Upon completion of data summary, a data table is obtained containing the features of interest (as rows) and its corresponding values per samples (as columns) suitable for further analysis. Data summary is classically performed before normalization, though exceptions exist (e.g. RMA).

For transcriptomic experiments, most often prior annotation information is used to obtain summary estimates. Microarray methods such as MAS5 and RMA use robust approaches and manufacturer annotation files to summarize probe level information into gene level expression estimates, whereas for RNA-seq data most often the number of aligned fragments per gene, transcript, exon… is used for further analysis. Software tools capable of RNA-seq data summary such as HTSeq (Anders *et al.* 2015) or featureCounts (Liao *et al.* 2017) therefore not only require SAM/BAM-files (or similar formats), but also annotation information regarding the genomic location and identifiers of genes, exons… which is typically provided by the user as a Gene Transfer Format (GTF) or the closely related more generic General Feature Format (GFF) file. These annotation files can be obtained from e.g. Ensembl (Hubbard *et al.* 2002), but may equally be in house created to target specific loci of interest. Rather than splitting up alignment and data summary in separate steps, pseudoaligners such as Kallisto provide direct quantification per transcript (provided in GTF files). As Kallisto is particularly fast and memory-efficient, it is frequently used without explicit focus on transcripts. In that case, the transcript “pseudocounts” per gene can be combined into single counts per gene (adjusted for transcript length) using specialized packages such as tximport (Soneson *et al.* 2015), compatible with common count-based statistical methods (see below).

For many applications the gene level (or related) is not readily relevant. For example, the same epigenetic tag may have different implications depending on genomic context [e.g. promoter DNA methylation is associated with gene silencing, exon methylation with active genes (Jones 2012)], and alternative units for data summary are required. For ChIP-seq and related enrichment data, e.g. from Cut&Tag or ATAC-seq experiments, most often peak calling algorithms are applied that allow one to identify regions of the genome for which the number of aligned reads is significantly higher than the background (i.e. “enriched”). Ideally, the background is estimated using a control sample (e.g. the DNA sample without the immunoprecipitation step), though several algorithms, e.g. MACS (Zhang *et al.* 2008) (model-based analysis of ChIP-seq), allow for estimation of the background using the actual ChIPped sample. These tools aim at identifying peaks/enrichment in single samples (with or without control), yet may also be used on in *silico* merged samples to delineate peaks (compared to e.g. a pool of controls). Identified peaks can then be summarized in a GFF file and provided to featureCounts or similar to perform data summary per sample. Such an approach leads to a uniform set of variables for subsequent statistical analysis and is more accurate and sensitive than peak calling on a sample-per-sample basis followed by an arbitrary peak merge step. Nevertheless, one should be aware of the fact that unbalanced sequencing depths or designs (e.g. far more cases than controls) may lead to the disappearance of interesting peaks. These problems have led to the development of specialized tools and metrics for peak detection with biological replicates (Landt *et al.* 2012, Zhang *et al.* 2014).

For bisulfite sequencing based DNA methylation analysis consensus for the optimal feature for summarization is lacking. Many analyses are performed at the CpG (or even cytosine) level (Feng *et al.* 2014), given that the methylation status of individual CpGs may be sufficient to have specific effects (Nile *et al.* 2008, Lian *et al.* 2022). More recently however, correlation between methylation levels of adjacent CpGs is used as a criterion to merge the latter for the statistical identification of “differentially methylated regions” (Hebestreit *et al.* 2013). Also for the related Infinium HumanMethylation beadarrays, CpG oriented statistical analysis is the standard approach, yet, region-based methylation analysis is not uncommon (Wang *et al.* 2012).

Finally, for single-cell analyses, typically an additional summarization step is performed to group cells per cell type, although also cell-level preprocessing, normalization (Brennecke *et al.* 2013) and differential analyses (Kharchenko *et al.* 2014, Buettner *et al.* 2015) methods exist. This grouping is typically established by clustering cells after dimensionality reduction (e.g. principal component analysis, PCA, and/or uniform manifold approximation and projection, UMAP, or t-distributed stochastic neighbor embedding, t-SNE), with subsequent identification of cell type by marker gene expression or through the use of a “reference atlas” [e.g. for mouse gastrulation (Pijuan-Sala *et al.* 2019)]. By subsequently aggregating the features of interest (e.g. expression, chromatin accessibility) over all cells of a given cell type for each sample separately, differential analysis becomes possible between cell types (clusters) or between conditions/samples (Tung *et al.* 2017). This is often referred to as a pseudobulk approach and these data can be further processed using the generic workflow outlined in this manuscript. Note that more specific single-analyses, such as the identification of cellular differentiation trajectories, are not further discussed here as they do not fit in the general framework of this manuscript.

### 2.3 Normalization (Fig. 1C)

Prior to statistical analysis, normalization is performed with the goal of making samples comparable to each other (thus normalization is performed on the columns of your data table). This is achieved by removal of consistent technical variation between samples, e.g. due to different sequencing library sizes or higher microarray probe intensities for some samples. Here, we do not consider intrasample normalization (e.g. to adjust for assay type in Infinium HumanMethylation arrays, or for gene length in RNA-seq data), as in molecular biology research one typically aims at comparing the same feature between samples, not between features within the same sample. Supplementary Table S1 contains an overview on the normalization strategies discussed and examples provided in the following paragraphs. Importantly, all normalization methods make assumptions, these are provided in Supplementary Table S1 as well.

The RMA preprocessing pipeline, e.g. assumes identical intensity distributions for all microarray samples. To this end, it uses quantile normalization (at the probe level), meaning that all values for a sample are replaced by values of a reference sample (e.g. average over all samples), matched on the corresponding ranks (e.g. highest value for sample of interest is replaced by highest value of reference sample, second highest by second highest and so on) (Bolstad *et al.* 2003). Unless ties are present (e.g. in count data, or due to saturation of microarrays) quantile normalization results in the identical distributions aimed for.

More widely used, typically for sequencing applications, but also e.g. by MAS5, is scaling normalization, where all values of a sample are multiplied by a sample-specific scaling factor. For example, library size normalization for sequencing experiments defines the scaling factor in such a way that upon normalization the sum of all counts (“library size”) is identical between all samples. Similar to quantile normalization, this assumes that the feature of interest (expression, methylation, …) is “on average” equally present in all samples. Knowledge of the underlying biology is important to evaluate whether this assumption is realistic or not. More advanced methods, e.g. TMM (trimmed-mean of M-values) (Robinson and Oshlack 2010), remove loci featured by high variation between samples when calculating scaling factors, as these typically include the differentially expressed (methylated, …) loci of interest which may compromise the normalization assumptions.

For epigenetic analyses, one should be particularly aware of the impact of normalization, given the often consistent differences between cases and controls. For example, the fact that many tumors are featured by general hypomethylation (Feinberg and Vogelstein 1983, Hon *et al.* 2012, Zhang *et al.* 2020) violates the assumptions of equal presence or equal distributions of the epigenetic feature of interest. For DNA methylation studies based on bisulfite treatment, normalization is less problematic, given that both methylated and unmethylated cytosines are measured within the same sample, leading to methylation percentages that are inherently normalized. This is also the case for Infinium HumanMethylation beadarrays, where additional normalization is possible (and based on rather robust assumptions), yet only leads to minor improvement in accuracy (Wu *et al.* 2014).

For DNA methylation studies based on affinity or immunoprecipitation, and for other enrichment based epigenomic studies (inc. assays for open chromatin), normalization is however less trivial. Note that for peak detection algorithms, normalization is already required when total input control samples (i.e. the same sample without enrichment procedure) are used. For the latter, library size normalization is typically applied, e.g. by MACS, yet as this procedure is rather conservative (inflation of solely background signal in control sample), candidate peak regions are often removed prior to normalization (Zhang *et al.* 2008, Rozowsky *et al.* 2009). On the other hand, when aiming at normalization *between* different (non-input control)samples for quantitative comparisons (e.g. disease cases versus healthy controls), using the background may be inappropriate due to varying signal-to-noise ratios between samples caused by experimental variability. ChIP-seq (and related) normalization procedures therefore often rely on exactly the opposite, i.e. they aim at inferring normalization factors from peaks that are common between samples (Shao *et al.* 2012).

Finally, it should be noted that specialized methods developed for RNA-seq data analysis (e.g. TMM) are also often applied on enrichment data. Yet, once again, one should be wary of the fact that many normalization methods may obscure genome-wide shifts in the epigenomic feature of interest. An option is to evaluate the presence of such genome-wide differences prior to the omics experiment, as can e.g. be done for DNA methylation by measuring the global genomic methylcytosine frequency per sample (Beck and Rakyan 2008). Given these complications, also experimental adaptations to the omics protocols—such as spiking in foreign material for referencing (Bonhoure *et al.* 2014)—have been developed as an alternative strategy.

Though specific QC metrics and plots exist for each preprocessing step (data type specific, data summary, normalization), these are typically combined as a single QC (QC2) upon completion of the full preprocessing pipeline and mostly rely on the same tools as for raw data QC. Besides a similar goal as QC1 (identification of aberrant samples), this QC step particularly aims to identify issues that arise during one of the preprocessing step, e.g. poor alignment for sequencing data, imperfect normalization, etc. (examples can be found in Supplementary Fig. S1D–H).

### 2.4 Hypothesis testing strategies (Fig. 1D)

Upon adequate (pre)processing, the data table can now be used for statistical analysis. The set of hypotheses to test highly depends on the research question for which the omics data was collected, however very often it comes down to finding loci where the measured features (e.g. genes, CpGs…) shows significant differences (e.g. in expression, methylation…) between two (or more) groups (e.g. case vs. control), hence often referred to as differential analysis. Hypothesis testing is thus performed for each row of your data table. For the continuation of this section we will consider the most simple example, a differential analysis of a given feature between two groups, but note that the concepts also hold for more complex contrasts. For microarrays, data are typically already normalized (see previous section) and log-transformed, thus *de facto* continuous and approximately Gaussian data readily suitable for statistical testing. For sequencing data, however, the raw—i.e. not yet normalized—count data are often modeled directly as a negative binomial distribution (see below), with the estimated normalization (i.e. scaling) factors per sample introduced in the statistical models as constants (Robinson and Oshlack 2010).

In many cases, standard statistical models [e.g. general(ized) linear models] for univariate analysis can be used, both for microarray and sequencing data. Yet, the often low number of replicates combined with an extremely high number of variables has led to specific adaptations for the omics field. These adaptations are mainly focused on accurate (within-group) variance estimation as relevant test statistics are typically inversely proportional with this variance (e.g. common independent t-statistic, $t=(\bar{X}-\bar{Y})/(S*\sqrt{\left(1/n\right)+(1/m)\;})$, with *X* and *Y* samples with resp. *n* and *m* observations and *S* the pooled variance for *X* and *Y*). Indeed, for microarray data, it was observed that many results obtained from standard tests were false positives due to underestimation of the variance for these “significant” loci. For example, suppose one uses a t-test to study the difference between two groups of samples. If tens of thousands (or more) of loci are analyzed with a low number of replicates, some loci will exhibit very low variance merely by chance, leading to high absolute values of the t-statistic and hence significant results even if the difference in mean between both groups is irrelevant. Methods such as SAM (Tusher *et al.* 2001) (significance analysis of microarrays) therefore implemented the concept of moderation: by evaluating all loci of the microarray, the general (“common”) variance is estimated, which is combined with the estimated variance for the individual locus to yield a more robust “moderated” variance estimate for that locus. SAM then generates a modified t-statistic using the moderated rather than the locus specific variance estimate, and permutations are used to test whether this modified statistic is sufficiently high or low to indicate significance. The developers of limma (Smyth 2004) (linear models for microarray analysis) took this one major step further and fitted this concept in a formal statistical framework. By calculating and attributing the degrees of freedom for both locus specific and common variance, they obtained a modified t-statistic that allows for formal inference. The latter approach did not only eliminate the need for permutations, thereby solving the low resolution problem for low numbers of replicates, but also exhibited an increase in power due to the fact that information is borrowed from the other loci (higher total degrees of freedom). Note that moderation is not confined to t-tests, as it can also be implemented in e.g. generalized linear models, and is not always necessary, e.g. when the number of biological replicates is sufficiently high.

Upon the advent of RNA-seq and related quantitative sequencing applications (e.g. ChIP-seq), the concept of moderation was adapted to the new setting. Whereas (expression) microarray data is typically more or less normally distributed after log-transformation, standard RNA-seq preprocessing yields count data (and associated scaling normalization factors). The original approach was to model these counts directly, most often by means of a negative binomial distribution as implemented in the widely used EdgeR and DESeq methods (Anders *et al.* 2013). The negative binomial distribution is in fact very similar to the Poisson distribution, which is commonly used to model count data. However, a Poisson distribution assumes that the variance equals the mean of a sample, which is only the case for technical variance, i.e. the variance caused when the same sample is sequenced multiple times. The negative binomial distribution therefore includes an additional variance parameter that enables to also capture biological variance. This negative binomial model is also applicable for pseudobulk single-cell data, or even for single-cell level data, though some modifications are required to accommodate for the latter’s high frequency of dropout events (excess zeroes) (Van Den Berge *et al.* 2018). A somewhat more recent strategy skips the rather complex negative binomial model and relies instead on log-transforming and normalizing (typically using TMM) the count data to obtain normal-like data suitable for an adapted version of limma (see next paragraph) (Law *et al.* 2014).

Indeed, independent of the data distribution, also the moderation methodology had to be adapted for sequencing data: contrasting microarray data, the variance for each locus appeared to depend on the average expression value for that locus. As a result, moderation for sequencing applications does typically not rely on the common variance over all loci, but on the “trended” variance, i.e. the empirical relationship between variance and mean of the expression values over all loci. The final moderated variance estimate per locus is then a compromise between the locus specific variance estimate and the predicted variance based on the average expression of the locus. Different omics methods such as DESeq2 (Love *et al.* 2014) and EdgeR (Robinson *et al.* 2009) use different approaches (either parametrically or non-parametrically) to obtain the predicted variance and the compromise.

Taking it one step further, limma voom—a limma extension for sequencing data—uses this type of trendline to predict the variances (of log-transformed and normalized counts) for each individual observation (i.e. sample-locus combination). These variances are subsequently used as “precision” weights in the limma model also used for microarray data. Though the transformed count data is not perfectly suited for limma (which assumes normality), this disadvantage is putatively outweighed by more accurate moderation. More specifically, by allowing for a varying impact of moderation per sample (even for the same locus), this method is particularly robust against (large) library size variation between samples. Finally, it should be noted that the practical use of moderation extends beyond robust variance estimation, as it has e.g. also been applied to obtain more robust estimates of log fold-changes for count data, which is particularly relevant for loci featured by generally low counts and/or outliers (Love *et al.* 2014).

Statistical inference for epigenomic data largely fits in the outlined RNA-seq framework, particularly for ChIP-seq and related enrichment data. For DNA methylation studies based on bisulfite sequencing though, additional modifications to the workflow are required. Infinium HumanMethylation results e.g. are typically expressed as β-values, i.e. the percentages of methylation per CpG, which follow a bimodal distribution [i.e. most loci are featured by either (almost) complete presence or absence of methylation]. Transforming these data to M-values, essentially the logarithm of the methylated/unmethylated probe intensity ratio, leads to data that is more complicated to be interpreted by humans, yet more suitable for statistical analysis using moderation compatible omics tools (Du *et al.* 2010, Wilhelm-Benartzi *et al.* 2013). One may use the same approach for bisulfite sequencing data [e.g. RnBeads (Assenov *et al.* 2014)], yet alternative methods exist that avoid calculating M-values, e.g. by means of beta-binomial models (Hebestreit *et al.* 2013), or the specialized use of EdgeR (Smyth *et al.* 2017). The underlying rationale for the latter methods is that identical M-values for a given CpG may be featured by different variances (resolution) due to varying sequencing depths between samples.

Upon completion of statistical analysis, a last QC step is performed. This QC step typically involves sanity checks of results, e.g. boxplots of top results to identify false positives due to outliers, and/or *post hoc* verification of assumptions to eliminate possible biases or to improve the filtering strategy (see further), e.g. *P*-value distribution (Supplementary Fig. S1I–J).

### 2.5 Hypothesis testing: balancing false discovery with power and robustness (Figs 1D and 2)

Independent of the used methodology, omics research typically entails hypothesis testing of tens to hundreds of thousands of features, leading to many false positive results if one would not adjust for multiple testing.

Error control through the commonly used family-wise error rate (FWER) methods such as Bonferroni tries to keep the chance of a single false positive result below a certain critical significance threshold. For example at an FWER of 0.05, the probability of having any false positive results rejecting the null hypothesis is controlled at 0.05. This is however overly conservative for omics experiments, as it will lead to a large fraction of missed but relevant results. Moreover, in omics experiments, a few false discoveries are acceptable, as main results are typically further validated. Therefore, error control is commonly performed through estimation of the false discovery rate (FDR), i.e. the fraction of false positive results among the list of positives (Storey and Tibshirani 2003). For example at an FDR of 0.05, 5% of statistical test results rejecting the null hypothesis are expected to correspond to false positives.

**Figure 2.**
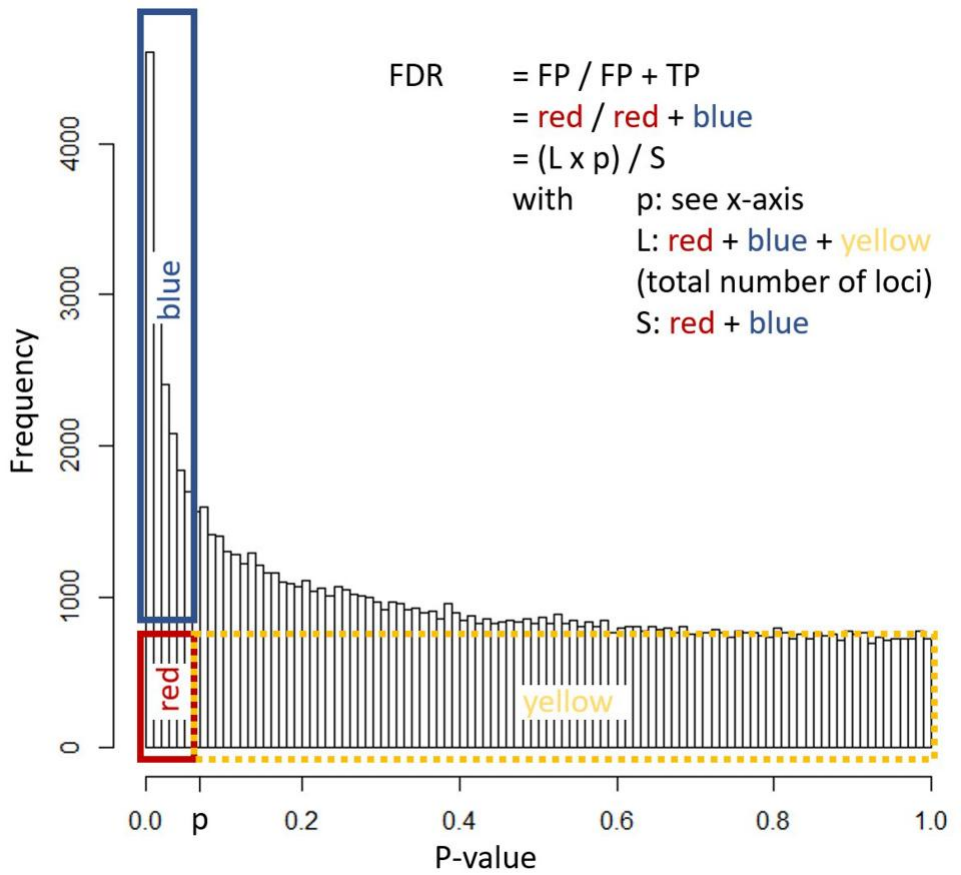
Intuitive interpretation of the Benjamini–Hochberg procedure. Under the null hypothesis and several assumptions (see text), we expect the *P*-value distribution to be uniform (yellow + red). For a certain *P*-value cutoff (*P*), in this case 0.05, the number of expected significant loci is determined (red) and divided by the observed amount of significant loci, S, (blue + red). In other words, we divide the expected false positives (FP, L*p) by the amount of observed positives, consisting of false positives and true positives (TP) (FP + TP, S) resulting in the false discovery rate (FDR).

The most commonly used procedure is the Benjamini-Hochberg FDR method, which is based on the crucial notion that under the null hypothesis (i.e. no association between locus and sample feature of interest) and several assumptions (e.g. continuous data, asymptotic approximation of used tests holds), *P*-values should follow by definition an (approximately) uniform distribution. If for a set of loci there is an association with the sample feature of interest, there will be an excess number of loci with lower *P*-values (see e.g. Fig. 2). Therefore, for any *P*-value cut-off, one does not only know the number of positives, but one can also estimate the expected number of false positive results under the null hypothesis as the fraction of the number of loci under study. The FDR corresponding to any *P*-value cut-off can hence be estimated as the ratio between both, and a final cut-off can be selected based on the predefined FDR (Fig. 2) (Benjamini and Hochberg 1995). Note that this method assumes that all loci are false positives when calculating the expected number of false positives. A more advanced class of procedures, collectively coined “local FDR” (Strimmer 2008), empirically estimates the actual number of loci following the null hypothesis for a given cutoff. Despite a more complicated procedure, this often leads to lower FDR estimates, i.e. more power.

Having sufficient power to find at least the most important effects in a dataset is indeed a major concern for omics experiments, given the often limited experimental designs (due to cost or sample availability). As stated higher, for this type of designs, moderation is particularly relevant, as it allows to borrow information from other loci, leading to more robust estimates and increased power. However, there are additional strategies to obtain these goals, e.g. by (i) data filtering, (ii) sample quality weighting, (iii) read trimming, or (iv) using larger units for analysis.

Data filtering refers to the removal of variables that are very unlikely to lead to significant and/or (biologically) relevant results, thereby leading to lower false discovery rates for actually relevant results. Variable filtering is often based on a biological rationale, e.g. DNA methylation studies solely focusing on CpG dinucleotides (filtering out CHG and CHH-methylation) or on QC properties, e.g. removal of Infinium HumanMethylation probes featured by known SNPs (Daca-Roszak *et al.* 2015). However, the largest impact on power is typically observed when filtering out non-informative loci, e.g. with too low intensities (microarrays), counts (sequencing) or methylation degrees (bisulfite data). Filtering is e.g. performed by default in the DESeq2 package for sequencing count analysis (Love *et al.* 2014). Moreover, removing these loci may also improve modeling of the mean-variance trend for sequencing data. Attention should however be paid to the exact filtering implementation, given that loci featured by low values in only a subset of samples may be exactly those loci of interest (e.g. silenced in the set of case samples). To guide the evaluation of filtering criteria for non-informative loci, one can use histograms or density plots of the obtained (unadjusted) *P*-values (cf Fig. 2) as these loci will show a peak near *P* = 1. Adequate filtering will remove the latter peak and again lead to a uniform distribution (except for the putatively significant results near *P* = 0, which should be ignored during this evaluation). Evaluating the *P*-value distribution prior to multiple testing is therefore a good practice, and even enables the detection of other potential problems, e.g. relating to assumptions. When filtering based on a biological rationale, it is possible that only few remaining loci remain. Then it is an option to analyze the full (or non-informative variable filtered) dataset to obtain (moderated) inference statistics per locus, followed by the filtering step and (re)calculation of the false discovery rates based on the *P*-values for the specific subset of interest. Nevertheless, it is crucial to mention that one should refrain from *P*-value hacking during filtering, i.e. criteria cannot be based on preliminary statistical testing results or on phenotypic data related to one’s research hypothesis. Finally, also novel strategies are emerging that attempt to avoid the hard filtering cut-offs, e.g. the use of data-driven hypothesis (i.c. locus) weighting (Ignatiadis *et al.* 2016).

Another strategy to improve power and robustness with limited study designs is to estimate sample quality and to use the latter as sample quality weights during statistical analysis, implemented e.g. in limma (Liu *et al.* 2015). This approach not only avoids removal of lower quality samples in settings with low numbers of replicates, but also gives more weight to more reliable data during statistical analysis. Power is however not only affected by the statistical procedures themselves, but also by the quality and nature of the input data. Read trimming e.g. only applies to sequencing data and entails removal of low quality or artificial (e.g. remaining sequencing adapters) parts of reads. As a consequence, more reads can be aligned due to lower numbers of mismatches, leading to a higher signal-to-noise ratio and thus more power (Bolger *et al.* 2014). Nevertheless, the use of aligners that allow for “soft-clipping”, i.e. masking of non-informative (e.g. adapter sequences) or low quality parts of a read, has been reported to lead to comparable or even better results (Liao and Shi 2020). Finally, the use of larger units for data summary (e.g. genes rather than exons or individual probes, methylated regions rather than individual CpGs) will lead to higher power due to overall larger values (sequencing) or a higher signal-to-noise ratio/lower impact of artefacts (arrays), yet at the cost of a lower resolution. Finally, it should not be forgotten that these strategies are in fact a poor man’s solution: including more biological replicates is still the better option to increase power and independent validation is a far better means to verify the “significance” of results.

## 3 Discussion and conclusions

Throughout this manuscript, we provided a conceptual overview of the generic omics data analytical workflow, more specifically for transcriptomic and epigenomic data, obtained from both array and sequencing based experiments. Though we did not aim for a comprehensive overview of individual methods, the shared characteristics of problems and solutions outlined here should enable one to appreciate the reasoning and strategies behind other data analytical approaches in the field. Nevertheless, for a more in depth overview of best practices, QC and/or critical choices for specific data analysis, we refer to specialized literature (see Introduction). Given the wide applicability of our data analytical flow, we enable and encourage readers to use insights gained from a specific analysis or data type and apply them conceptually to other analyses. Moreover, the common pitfalls and (statistical) strategies discussed in the current generic framework can be extrapolated to a plethora of new and emerging technologies (provided appropriate additional preprocessing). Thus rendering the our workflow also applicable to emerging technologies such as SOMAscan data (microarray-like data in proteomics) (Gold *et al.* 2010), single molecule sequencing [e.g. Oxford Nanopore sequencing (Jain *et al.* 2016)], spatial transcriptomics (Ståhl *et al.* 2016) or even technologies yet to be designed.

The conceptual aims of this manuscript come with a few important drawbacks, and the generic pipeline introduced should therefore not be considered as a “gold standard”. First, several data analytical options were not considered as they are too data type specific. As mentioned earlier, researchers should be particularly wary of potential normalization problems, particularly for epigenomic data analysis. For statistical analysis, we focus on the limma pipeline as this well-developed package can be applied on broadly different data types (microarrays and sequencing, expression and epigenomic data), however in specific cases other software might be more suited. Finally, for single cell data, e.g. scATAC-seq or scRNAseq, our generic workflow mainly fits the pseudobulk strategy, though more dedicated methods are also available. Nonetheless, other approaches often rely on the same ground principles safeguarded in limma, e.g. moderation, variance modeling for sequencing data, etc. Finally, we would like to stress the fact that any omics workflow only leads to a promising set of candidate biomarkers for further large scale validation in independent samples (preferably using a different technology such as qRT-PCR).

In conclusion, a generic pipeline was presented to illustrate the major concepts (problems and solutions) of epigenomic and transcriptomic data analysis. Conceptual and statistical insights explained in this manuscript, allow readers familiar with omics technology to deepen their understanding of transcriptomic and epigenomic data analysis and facilitate progression to more specialized and expert methods and bioinformatics tools.

## Supplementary Material

vbae019_Supplementary_Data

## Data Availability

There are no new data associated with this article.
