# Correction to: Quantitative transcriptomic and epigenomic data analysis: a primer

**DOI:** 10.1093/bioadv/vbae091

**Published:** 2024-07-18

This is a correction to: Louis Coussement, Wim Van Criekinge, Tim De Meyer, Quantitative transcriptomic and epigenomic data analysis: a primer, *Bioinformatics Advances*, Volume 4, Issue 1, 2024, vbae019, https://doi.org/10.1093/bioadv/vbae019

When this article originally published, the files containing Supplementary Table 1, Supplementary Figure 1 and Supplementary Information S1 were omitted in error.

These supplementary files have been added to the article’s Supplementary data section.

## Supplementary Material

vbae091_Supplementary_Data

