# Supplementary material for: Quantitative transcriptomic and epigenomic data analysis: a primer
Source: Bioinform Adv. 2024 Feb 10;4(1):vbae019. doi: 10.1093/bioadv/vbae019 (PMC10997052; doi:10.1093/bioadv/vbae019)
Supplement: vbae019_Supplementary_Data [file vbae019_supplementary_data.zip › List_of_Supplementary_Tables_and_Figures.docx]

List of Supplementary Tables, Figures and Information

**Supplementary Table 1** – Overview of the subset of normalization methods discussed in this manuscript. We provide an overview of methods discussed including the assumption they make and their general applicability. Array stands for array-based technology and NGS for Next Generation Sequencing based technology.

**Supplementary Figure 1** – Examples of quality control (QC) plots for the different QC steps in Figure 1. **QC1**: data type specific QC on the “raw data” to identify batch effects or samples featuring artefacts. **(A)** Evaluation of spatial artefacts in microarray data suggests to exclude sample a from further analysis. **(B)** Evaluation of basecalling quality (indicated by “phred scores”) provides an overview of the average quality, and its variance, of sequenced nucleotides as a function of the read length. **(C)** The evaluation of average sequencing read GC content identifies a major batch effect largely paralleling the feature of interest, thereby possibly compromising further statistical analyses. QC typically also involves an evaluation of total read number, read lengths, … **QC2**: in addition to the aims outlined for QC1, this QC step also aims at evaluating the impact of preprocessing. **(D)** Density plots for raw intensity distributions of different array samples indicate deviating distributions for samples *a* (pink, bimodal, cf. (A)) and *f* (light blue, overall higher intensities). **(E)** Density plots for normalized intensity distributions indicates successful normalization (mostly equal distributions with appropriate shape), yet based on raw and normalized intensity profiles but also (A), sample *a* should be removed, whereas the quality of sample *f* should be further evaluated based on other QC metrics **(F)** Number of RNA-seq reads assigned to a feature (genes in this case), i.e. after alignment and data summary. Samples displaying a very low number of remaining attributed features can be excluded from downstream analysis, e.g. sample 9. **(G)** A multi-dimensional scaling plot (MDS-plot) is a dimensionality reduction plot and can be used to detect deviating samples, e.g. sample 16 (note the difference in scale of both axes, i.e. S28 and S26 are less deviating). **(H)** MA-plots, depicting log-fold-change between sample groups (or individual samples) as a function of average (log) expression, can be used to identify issues with normalization. Whereas the bulk of the data points should typically be centered around the logFC = 0 line (no difference in expression), it is shifted slightly upward, indicating ok but imperfect normalization. **QC3**: the main goal of QC after statistical analysis is *post hoc* confirmation of certain assumptions and a sanity check of results. **(I)** Under the null hypothesis (e.g. no differences between groups), one expects a uniform p-value distribution. With actual significant results, an enrichment for p-values close to zero should be observed. The presence of other peaks typically indicates the need for additional filtering (e.g. when present around 1 for sequencing data) or the presence of bias. **(J)** Boxplots can be constructed for the top results of statistical analysis, thus allowing to assess robustness of results (e.g. impact of outliers). Abbreviations used in figure: NGS, Next Generation Sequencing; %GC, GC content.

**Supplementary Information 1** – Omics data repositories.
