# Supplementary material for: Quantitative transcriptomic and epigenomic data analysis: a primer
Source: Bioinform Adv. 2024 Feb 10;4(1):vbae019. doi: 10.1093/bioadv/vbae019 (PMC10997052; doi:10.1093/bioadv/vbae019)
Supplement: vbae019_Supplementary_Data [file vbae019_supplementary_data.zip › SupplementaryInformation1.docx]

## Omics data repositories

The increase of omics applications in the field of molecular research is not only powered by ongoing technical and data analytical improvements, but also by the general “public availability” policy for omics data. Perhaps the most well-known example at the moment is the Sequence Read Archive (SRA) (Kodama et al., 2012), a concerted international effort between the National Center for Biotechnology Information (NCBI, Gene Expression Omnibus (GEO) repository (Clough and Barrett, 2016)), the European Bioinformatics Institute (EBI, ArrayExpress repository (Kolesnikov et al., 2015)) and the DNA Data Bank of Japan (DDBJ) (Mashima et al., 2016) to create a repository providing both raw sequencing data as well as processed data. Moreover, ArrayExpress, GEO and DDBJ do not only provide public sequencing data, they also store microarray and related omics datasets. Nevertheless, particularly the immense volumes of sequencing data are challenging, explaining the joint SRA effort to manage this data type (Kodama et al., 2012).

These and similar initiatives to preserve omics data and making them publicly available are crucial to the field: the large cost of data generation combined with the observation that typically only few hypotheses are explored per dataset are powerful incentives to reuse the data for alternative purposes, e.g. the analyses performed in a next part of this manuscript. Moreover, it allows to verify published results. Consequently, many prominent journals require to make omics data available as condition for publication of results. However, in the case of identifiable (i.e. SNP containing) human data, controlled access is warranted - and often even legally obligatory – due to privacy reasons. Therefore, controlled access platforms such as dbGaP (the database of Genotypes and Phenotypes; NCBI) (Tryka et al., 2014) and EGA (the European Genome-Phenome Archive; EBI) (Lappalainen et al., 2015) facilitate monitored sharing of (possibly) sensitive data, and evaluate the research goals and researchers’/institution’s background prior to giving access to the data. Finally, also several large-scale omics projects (both bulk and single cell sequencing) make their data publicly available through such platforms, e.g. The Cancer Genome Atlas (TCGA) & the Genotype-Tissue Expression project (GTEx) through dbGaP, the Human Cell Atlas (HCA) through multiple platforms (EBI, Broad Institute, UCSC), whereas others predominantly host their own large-scale data (e.g. ENCODE project). See Table 1 for a more elaborate overview of omics data repositories. Here we focused on data repositories with quantitative transcriptomic and epigenomic data, as genomic data repositories (e.g. 1000 Genomes project (Auton et al., 2015), GnomAD (Karczewski et al., 2020), Biobank UK (Sudlow et al., 2015)) are less relevant for the workflow we present.

**Table 1** - Summary of important quantitative omics data resources/repositories. Numbers of samples were obtained on December 16^th^, 2022.

| **Name** | **Type of data** | **# Samples*** | **Website** |
| --- | --- | --- | --- |
| SRA  (Kodama et al., 2012) | Raw reads (+ alignment information; sequence data) | NA | https://www.ncbi.nlm.nih.gov/sra |
| ArrayExpress  (Kolesnikov et al., 2015) | Processed data (sequencing & array) | NA | https://www.ebi.ac.uk/arrayexpress |
| GEO  (Clough and Barrett, 2016) | Processed data (sequencing & array) | NA | https://www.ncbi.nlm.nih.gov/geo/ |
| ENCODE  (Feingold et al., 2004) | Genomic, epigenomic and transcriptomic data for the identification of functional elements in the human genome | 12,667  (human) | https://www.encodeproject.org/ |
| TCGA  (Weinstein et al., 2013) | Genomic, epigenomic and transcriptional data for (human) cancer tissues and associated controls (sequencing & array) | 19,334 | https://cancergenome.nih.gov/ |
| ICGC  (Hudson et al., 2010) | Genomic, epigenomic and transcriptional data for (human) cancer tissues and associated controls (sequencing & array) | 24,289 | http://icgc.org/ |
| GTEx  (Lonsdale et al., 2013) | Tissue specific expression data, subject genotypes (sequencing & array) | 17,382 (from 948 donors) | https://gtexportal.org/home/ |
| HCA | Cell type specific transcriptomic and epigenomic data | >34M cells (from 4,800 donors) | https://data.humancellatlas.org/ |
| dbGAP  (Tryka et al., 2014) | Restricted access omics data | NA | https://www.ncbi.nlm.nih.gov/gap |
| EGA  (Lappalainen et al., 2015) | Restricted access omics data | NA | <https://www.ebi.ac.uk/ega/home> |

*Number of (human) samples in the project, NA: not applicable (community driven repositories rather than large-scale individual omics projects)

## References

Auton, A., Abecasis, G. R., Altshuler, D. M., Durbin, R. M., Abecasis, G. R., Bentley, D. R., et al. (2015). A global reference for human genetic variation. *Nature* 526, 68–74. doi: 10.1038/nature15393.

Clough, E., and Barrett, T. (2016). The Gene Expression Omnibus database. *Methods Mol. Biol.* 1418, 93–110. doi: 10.1007/978-1-4939-3578-9_5.

Feingold, E. A., Good, P. J., Guyer, M. S., Kamholz, S., Liefer, L., Wetterstrand, K., et al. (2004). The ENCODE (ENCyclopedia of DNA Elements) Project. *Science* 306, 636–640. doi: 10.1126/science.1105136.

Hudson, T. J., Anderson, W., Aretz, A., Barker, A. D., Bell, C., Bernabé, R. R., et al. (2010). International network of cancer genome projects. *Nature* 464, 993–998. doi: 10.1038/nature08987.

Karczewski, K. J., Francioli, L. C., Tiao, G., Cummings, B. B., Alföldi, J., Wang, Q., et al. (2020). The mutational constraint spectrum quantified from variation in 141,456 humans. *Nature* 581, 434–443. doi: 10.1038/s41586-020-2308-7.

Kodama, Y., Shumway, M., and Leinonen, R. (2012). The sequence read archive: Explosive growth of sequencing data. *Nucleic Acids Res.* 40, 2011–2013. doi: 10.1093/nar/gkr854.

Kolesnikov, N., Hastings, E., Keays, M., Melnichuk, O., and Tang, Y. A. (2015). ArrayExpress update -simplifying data submissions. *Nucleic Acids Res.* 43, D1113–D1116. doi: 10.1093/nar/gku1057.

Lappalainen, I., Almeida-King, J., Kumanduri, V., Senf, A., Spalding, J. D., Ur-Rehman, S., et al. (2015). The European Genome-phenome Archive of human data consented for biomedical research. *Nat. Genet.* 47, 692–695. doi: 10.1038/ng.3312.

Lonsdale, J., Thomas, J., Salvatore, M., Phillips, R., Lo, E., Shad, S., et al. (2013). The Genotype-Tissue Expression (GTEx) project. *Nat. Genet.* 45, 580–585. doi: 10.1038/ng.2653.

Mashima, J., Kodama, Y., Kosuge, T., Fujisawa, T., Katayama, T., Nagasaki, H., et al. (2016). DNA data bank of Japan (DDBJ) progress report. *Nucleic Acids Res.* 44, D51–D57. doi: 10.1093/nar/gkv1105.

Sudlow, C., Gallacher, J., Allen, N., Beral, V., Burton, P., Danesh, J., et al. (2015). UK Biobank: An Open Access Resource for Identifying the Causes of a Wide Range of Complex Diseases of Middle and Old Age. *PLoS Med.* 12, 1–10. doi: 10.1371/journal.pmed.1001779.

Tryka, K. A., Hao, L., Sturcke, A., Jin, Y., Wang, Z. Y., Ziyabari, L., et al. (2014). NCBI’s database of genotypes and phenotypes: DbGaP. *Nucleic Acids Res.* 42, 975–979. doi: 10.1093/nar/gkt1211.

Weinstein, J. N., Collisson, E. A., Mills, G. B., Shaw, K. R. M., Ozenberger, B. A., Ellrott, K., et al. (2013). The cancer genome atlas pan-cancer analysis project. *Nat. Genet.* 45, 1113–1120. doi: 10.1038/ng.2764.
