# Supplementary material for: Quantitative transcriptomic and epigenomic data analysis: a primer
Source: Bioinform Adv. 2024 Feb 10;4(1):vbae019. doi: 10.1093/bioadv/vbae019 (PMC10997052; doi:10.1093/bioadv/vbae019)
Supplement: vbae019_Supplementary_Data [file vbae019_supplementary_data.zip › SupplementaryTable1.docx]

| **Normalisation methods** | **Examples** | **Assumption(s) made** | **Typically used in** |
| --- | --- | --- | --- |
| Quantile normalisation | RMA (Array) | Identical distributions between samples | Transcriptomics |
| Scaling normalization | MAS5 (Array) | Robust average intensities are the same between samples (excluding extreme values) | Transcriptomics |
|  | Library size norm. (NGS) | Total counts ("library size") of feature of interest (expression, modification…) are the same between samples | Transcriptomics,  ChIP-seq |
|  | TMM (NGS) | Upon removal of more variably expressed genes between samples, higher weights for more highly expressed genes leads to more robust scaling | Transcriptomics,  DNA methylation experiments,  ChIP-seq |
| Spike-in normalization | n.a. | Improved normalisation by using foreign material with a predefined concentration | Can be used if technology allows for it |
| n.a. = not applicable |  |  |  |
