# Supplementary figures and images for: Quantitative transcriptomic and epigenomic data analysis: a primer
Source: Bioinform Adv. 2024 Feb 10;4(1):vbae019. doi: 10.1093/bioadv/vbae019 (PMC10997052; doi:10.1093/bioadv/vbae019)

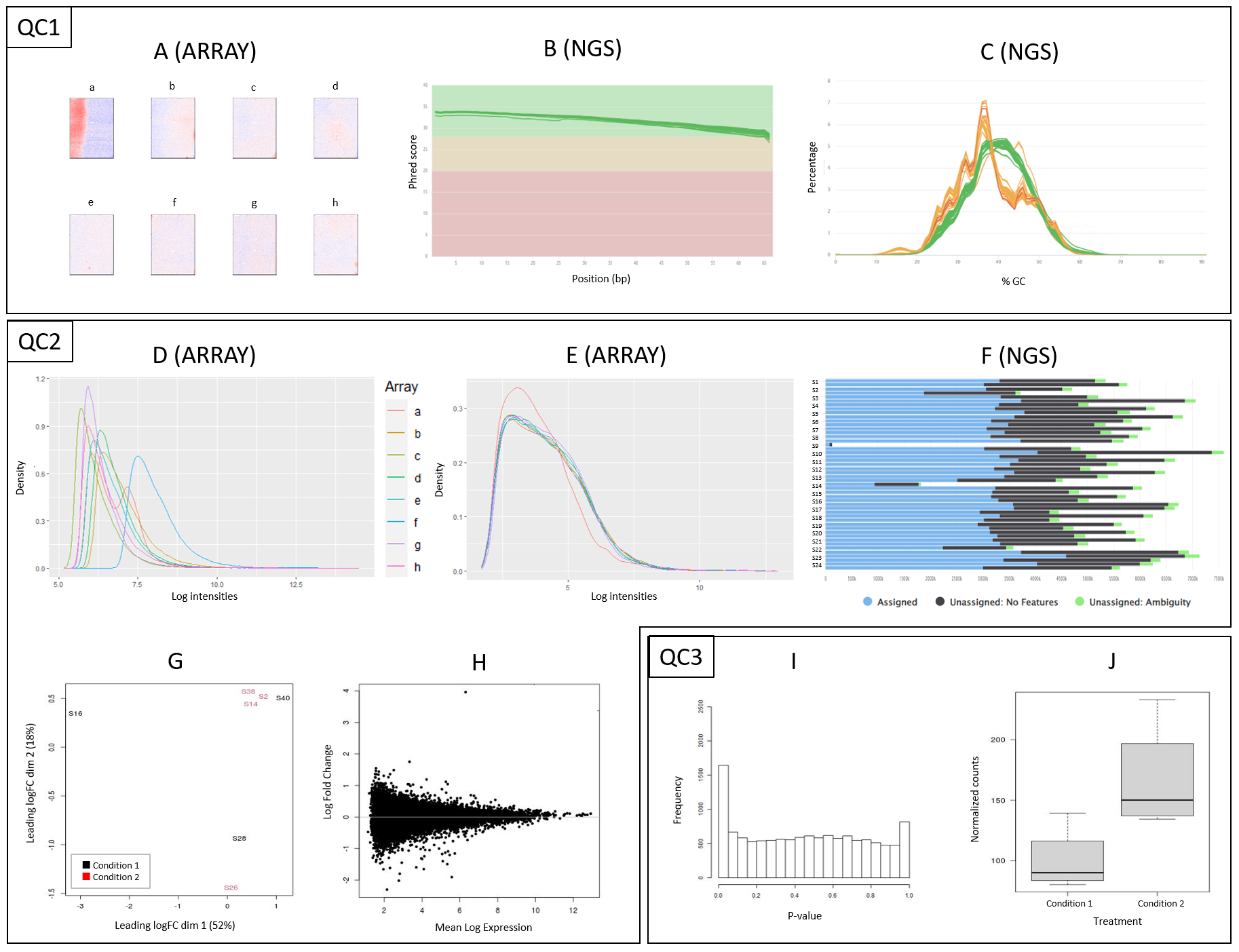

Supplement: vbae019_Supplementary_Data [file vbae019_supplementary_data.zip › SupplementaryFigure1.png]
